# New perforated radiation shield for anesthesiologists: Monte Carlo simulation of effects

**DOI:** 10.1093/jrr/rrac106

**Published:** 2023-01-25

**Authors:** Ayaka Yanagawa, Takeshi Takata, Taichi Onimaru, Takahiro Honjo, Takeyuki Sajima, Akihito Kakinuma, Akihisa Kataoka, Jun’ichi Kotoku

**Affiliations:** Department of Anesthesia, Teikyo University, Tokyo 173-8605, Japan; Advanced Comprehensive Research Organization, Teikyo University, Tokyo 173-8605, Japan; Department of Anesthesia, Teikyo University, Tokyo 173-8605, Japan; Department of Anesthesia, Teikyo University, Tokyo 173-8605, Japan; Department of Anesthesia, Teikyo University, Tokyo 173-8605, Japan; Department of Anesthesia, Teikyo University, Tokyo 173-8605, Japan; Division of Cardiology, Department of Internal Medicine, Teikyo University, Tokyo 173-8605, Japan; Graduate School of Medical Care and Technology, Teikyo University, Tokyo 173-8605, Japan

**Keywords:** anesthesiologist, protection, radiation, structural heart disease (SHD), shield, simulation

## Abstract

Catheterization for structural heart disease (SHD) requires fluoroscopic guidance, which exposes health care professionals to radiation exposure risk. Nevertheless, existing freestanding radiation shields for anesthesiologists are typically simple, uncomfortable rectangles. Therefore, we devised a new perforated radiation shield that allows anesthesiologists and echocardiographers to access a patient through its apertures during SHD catheterization. No report of the relevant literature has described the degree to which the anesthesiologist’s radiation dose can be reduced by installing radiation shields. For estimating whole-body doses to anesthesiologists and air dose distributions in the operating room, we used a Monte Carlo system for a rapid dose-estimation system used with interventional radiology. The simulations were performed under four conditions: no radiation shield, large apertures, small apertures and without apertures. With small apertures, the doses to the lens, waist and neck surfaces were found to be comparable to those of a protective plate without an aperture, indicating that our new radiation shield copes with radiation protection and work efficiency. To simulate the air-absorbed dose distribution, results indicated that a fan-shaped area of the dose rate decrease was generated in the area behind the shield, as seen from the tube sphere. For the aperture, radiation was found to wrap around the backside of the shield, even at a height that did not match the aperture height. The data presented herein are expected to be of interest to all anesthesiologists who might be involved in SHD catheterization. The data are also expected to enhance their understanding of radiation exposure protection.

## INTRODUCTION

Existing freestanding radiation shields for anesthesiologists in the operating room are typically simple rectangles. When working with a patient on the other side of the shield, the anesthesiologist must either move the shield or lean over it in an awkward position. This arrangement greatly impedes efficiency or radiation protection, or both. Moreover, the shields are not comfortable to use. Therefore, we devised a new ‘perforated radiation shield.’

The rapid spread of catheterization for cardiac diseases has led to development of the concept of structural heart disease (SHD) as a general term for the diseases it treats. Minimally invasive catheter-based therapies such as transcatheter aortic valve replacement (TAVR) and transcatheter edge-to-edge repair (TEER) are now available for SHDs such as aortic stenosis and mitral regurgitation, which had required open-heart surgery.

Catheterization requires fluoroscopic guidance. It therefore exposes health care professionals to radiation exposure risk. The American College of Cardiology recommend that health care professionals wear protective clothing such as aprons, vests, necks and glasses made of 0.25 mm or greater lead or lead equivalent during cardiovascular therapy using ionizing radiation, also strongly recommending additional portable shielding [1]. When used appropriately, these shields can reduce radiation doses to deficient levels [2]. Few reports have described installation of these devices at the patient’s head side, i.e. for the anesthesiologist [3–5]. As reasons, Mayr *et al.* stated that installing such shields would compromise access to the patient’s head and would compromise C-arm positioning [3].

Therefore, we have devised a new perforated radiation shield to protect anesthesiologists and echocardiographers, who, like anesthesiologists, are exposed to radiation at the patient’s head side. This freestanding board has work apertures on both the left and right sides.

Most of the radiation received by the anesthesiologist standing at the patient’s head is from Compton-scattered photons. No report has described how much the anesthesiologist’s radiation dose can be reduced by installing radiation shields. To estimate this reduction, a physics-based Monte Carlo simulation is fundamentally important, but such a simulation requires enormous computational resources.

For this study, we used the fast dose estimation system for interventional radiology (FDEIR) [6], a Monte Carlo system that Takata *et al.* developed, to estimate the whole-body doses to anesthesiologists and the air dose distribution in operating rooms with and without the new radiation shielding. The shielding effect differences depending on the aperture size were also examined.

## MATERIALS AND METHODS

**Fig. 1:**
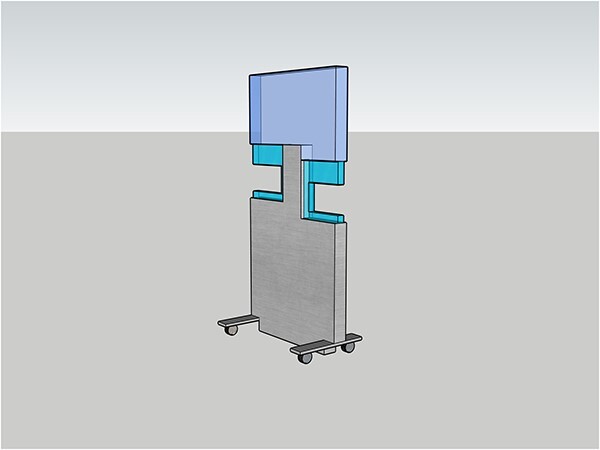
Our new perforated radiation shield is a freestanding radiation protective board with work apertures on both the left and right sides. The apertures height and size can be changed using the attached cartridge.

The shape of the radiation shield used for the simulation is portrayed in Fig. 1. It is about 10 cm wider than the commercially available protective boards. It is made of 1 mm lead or 1 mm lead equivalent acrylic. The aperture height and size can be changed using the attached cartridges.

The aperture size and height were determined to allow the worker’s arm to reach the patient’s mouth through the apertures. Anesthesiologists often work with both hands in a mid-back position while observing the patient. However, when performing transesophageal echocardiography, the echocardiographer is in a standing position with only the right arm extended into the aperture while checking the echo monitor. The echocardiographer’s movements are restricted. For this reason, the aperture on the echocardiographer’s side is more extensive and higher than on the anesthesiologist’s side.

Simulations were performed with the following four patterns: without the radiation shield, with the cartridge removed and large openings on both sides, with the cartridge and small openings on both sides, and with a completely rectangular radiation shield with no aperture at all.

### Simulation system

An FDEIR [6], which was used to estimate the exposure dose, simulates the radiation exposure dose in the diagnostic energy range using Monte Carlo method. Its accuracy was well validated in earlier comparative studies of dosimetry and other Monte Carlo codes [6–8].

The simulation was conducted on a single graphical processing unit (GPU; Tesla P100; NVIDIA Corp.) on a supercomputing system (SGI Rackable C2112-4GP3/C1102-GP8, Reedbush-L; Silicon Graphics International Corp.) at the Information Technology Center of the University of Tokyo. This study simulated a trillion incident photons with 5 keV cut-off energy for the photon. The simulation suppressed electron transport to accelerate the calculations.

### Factor of scaling on dose conversion

FDEIR calculates not the absolute dose, only the relative dose per number of incident photons. Therefore, we calculated a factor of scaling on dose conversion, which defines the number of photons as a per milliampere second value, to convert the simulated relative dose to absolute dose with FDEIR and a radiophotoluminescence (RPL) dosimeter (GD-352 M; Chiyoda Technol. Corp.) based on a reported method [6].

We set the geometry to obtain the factor of scaling on dose conversion (Supplementary Fig. 1). Two RPL dosimeters were placed at the center of the field of view in the water-equivalent phantom on the geometry. The doses were measured under 12 conditions while changing the source-to-surface distance (60–70 cm) and depth (0–15 cm). Other conditions were the same, as shown in Table 1.

**Table 1:** Fluoroscopic conditions obtained from our institution

Tube voltage	80 kV
mA	3.0
fps	3.75
Field of view	156.5 mm × 156.5 mm
Source to surface distance (at Posterior—Anterior direction)	60 cm
Source to image-receptor distance	100 cm
Filter	2.5 mmAl +0.4 mmCu +1.0 mmAl

For the simulation, after constructing a voxelized geometry resembling the measurement geometry (Supplementary Fig. 1), we simulated the dose under the same conditions as the measurement. Then, the relation between simulated doses was approximated using a linear function with a zero intercept [6]. The slope of the line was used as the factor of scaling on dose conversion.

### Simulation of anesthesiologist exposure

**Fig. 2:**
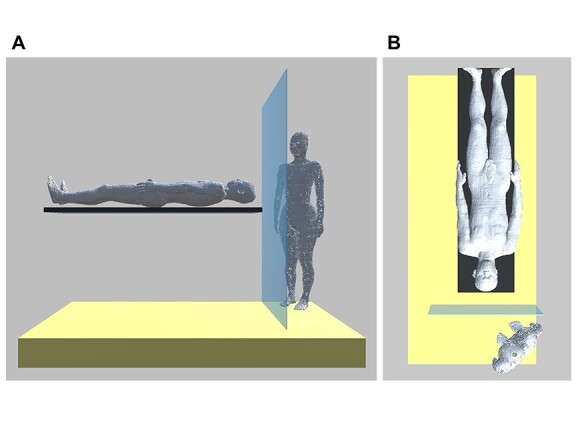
The anesthesiologist phantom is in a 45° oblique position to the patient phantom: A is the view from the patient’s left side; B is the view from above.

To estimate the dose to the anesthesiologist, we constructed a voxelized geometry divided into mesh voxels (2 mm^3^), resembling a cardiac catheterization suite at our institution. The simulation geometry was constructed from an undertable X-ray source, an imaging table, air, a concrete floor, a whole-body male model as a patient and a whole-body female model as an anesthesiologist (Fig. 2). The anesthesiologist model was transformed posturally to reflect an actual anesthesiologist posture faithfully [9].

We assigned mass density and compositions for the eye lens, bone and skin based on International Commission on Radiological Protection (ICRP) publication 110 [10]. Other tissues were treated as water or air for simplicity.

We particularly examined a situation related directly to the risk of radiation dermatitis during SHD procedures under the Posterior–Anterior (PA) projection, the Cusp-overlap projection, which is the right anterior oblique 9 and caudal 1 direction, the Perpendicular, which is the left anterior oblique 16 and cranial 13 direction. The PA projection is a primary fluoroscopic direction, which is almost always used during TEER procedures. The Cusp-overlap projection is used for TAVR self-expanding valves. The Perpendicular projection is used for balloon-expanding valves. Radiation is delivered from these angles for the longest time during the procedure. Fluoroscopic conditions are presented in Table 1. These conditions were determined based on a TEER procedure at our institution using fluoroscopic unity: Allura Xper FD20 (Koninklijke Philips N.V.).

### Simulation of air absorbed dose distribution

Simulations of air-absorbed dose distributions were conducted using FDEIR with a simple geometry that included the male patient model consisting of water, air and ordinary concrete walls of 20 cm thickness (8.1 m × 7.3 m × 3.4 m). The space was divided into mesh voxels (5 cm × 5 cm × 5 cm).

## RESULTS

### Factor of scaling on dose conversion

Supplementary Figure 2 presents measured and simulated doses with a least-squares fitting straight line for computing the factor of scaling on dose conversion. The linear regression equation for them is $y=1.10\ x$. The coefficient of determination, the square of Pearson’s correlation coefficient (*r* = 0.995), was 0.991. The result suggests that measured and simulated doses are proportional. Linear regression is plausible. For this study, we defined the line slope as the factor of scaling on dose conversion.

### Simulation of anesthesiologist’s exposure

**Fig. 3:**
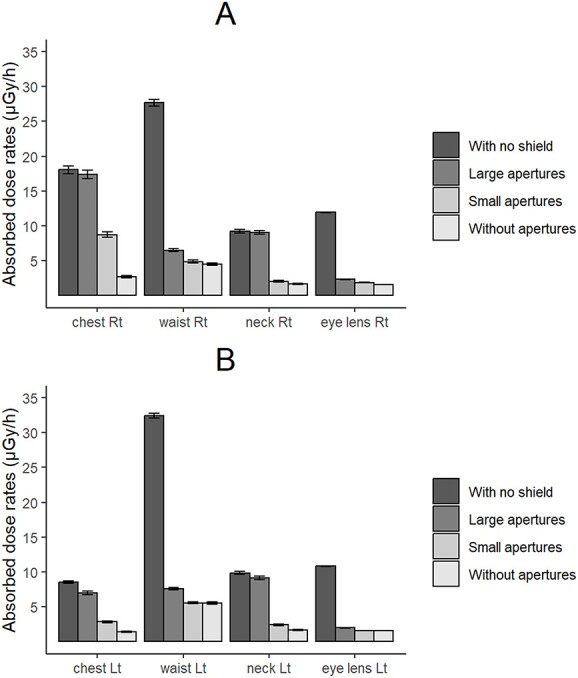
Absorbed dose rates for skin and lens (PA projection): A is the right side; B is the left side. Error bars show standard deviations (1σ) for simulations.

**Fig. 4:**
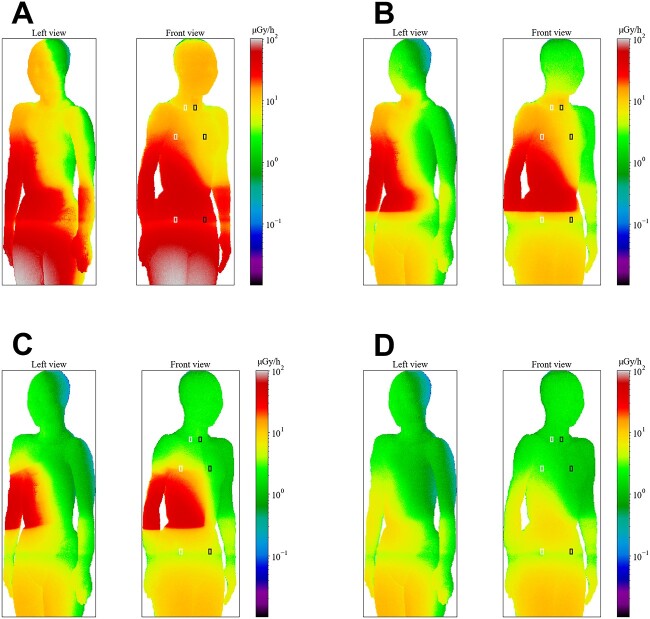
Skin dose rate distributions on the anesthesiologist’s body surface (PA projection).

The dose rates for the anesthesiologist’s body surface are presented in Fig. 3 and in Supplementary Figs 4 and 5. Figure 3 presents the dose rates for the PA projection. The dose rate distributions for the Cusp-overlap and Perpendicular projections are shown respectively in Supplementary Figs 4 and 5. Examples of skin dose rate distributions are shown in Fig. 4.

The dose rates for the eye lens and waist surfaces were reduced to approximately 20% or less when a radiation shield was installed, irrespective of the aperture size in the shield. On the chest and neck surfaces, doses differed depending on the aperture size, and depending on the neck surface. If the apertures were small, then the dose was reduced to a level comparable to that without an aperture (15% left, 15% right).

Supplementary Figure 3 shows the scattered photon trajectory obtained from the simulation, demonstrating that the photons are reflected off the operating table, patient and floor. It is apparent that most photons hitting the anesthesiologist are these Compton-scattered photons.

### Simulation of the air-absorbed dose distribution

**Fig. 5:**
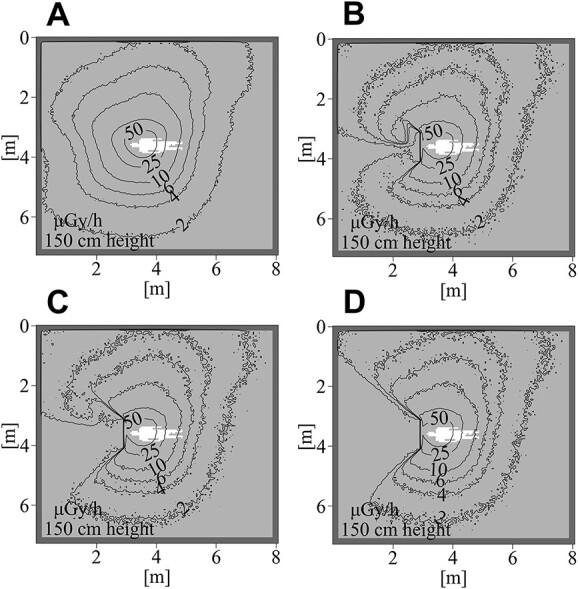
Air-absorbed dose distribution (PA projection). It is at 145–150 cm height from the floor and is intended for the eye lens height of an adult female phantom (160.8 cm).

The air dose distribution maps for each radiation shield’s installation and irradiation condition are shown in Fig. 5 and Supplementary Figs 6–8.

The air dose distribution map shown in Fig. 5 is for a 145–150 cm height from the floor. It incorporates consideration of the eye lens of an adult female phantom (160.8 cm). The fluoroscopy direction is the PA projection.

Supplementary Figure 6 shows the height distribution of the PA projection with other radiation-sensitive organs, i.e. thyroid (130–135 cm) and ovaries (80–85 cm). Supplementary Figures 7 and 8 respectively show the Cusp-overlap projection and Cusp-overlap projection.

Without a radiation shield, the dose rate decreased with distance from the tube. When the radiation shield was installed, results showed that a fan-shaped area of dose rate decrease was generated in the area behind the shield, as seen from the tube sphere.

The heights of the apertures from the floor are, for large aperture, 100–140 (cm) for the echocardiographic side and 90–130 (cm) for the anesthetic side. For the small aperture, they are 105–125 (cm) for the echocardiographic side and 95–115 (cm) for the anesthetic side. Not one of the heights corresponds to the radiation shield aperture, but in the case with the aperture, radiation was detected as wrapping around the backside of the shield. This finding was more pronounced in the case of a large aperture than for a small aperture.

These trends found for the PA projection were observed in the Cusp-overlap and Perpendicular projections (Supplementary Figs 4–7). We emphasize differences attributable to the irradiation direction, in the simulation of the anesthesiologist’s exposure, exposure to the chest and waist surfaces were highest in the PA projection, where the tube sphere was facing straight up. Exposure to the eye lens was highest in the Cusp-overlap projection, where radiation from the tube sphere faces the patient’s head.

Regarding spatial dose distribution, the high-dose area shifted to the right in the Cusp-overlap projection and to the left in the Perpendicular projection compared to the PA projection. In both cases, the area above 50 μGy / hr was expanded at the risk organ height.

## DISCUSSION

For the first time ever reported, we simulated the whole-body dose to anesthesiologists during SHD catheterization and simulated the air dose distribution in a hybrid operating room using an FDEIR Monte Carlo calculation system. For this simulation, we examined the protective effect of the perforated radiation shield, which we developed. Then we compared the difference in doses depending on the aperture size. One can reasonably expect that a larger radiation shield aperture makes it easier to perform the work. By contrast, a smaller aperture gives a lower exposure dose. For this simulation, the difference in exposure doses to the lens and the waist surface was small depending on the aperture size. The exposure dose to the neck surface increased when the aperture was large, but it was comparable to that without the aperture when the aperture was small. However, the dose at the neck surface increases with the aperture size. The aperture size is set at a comfortable size for anesthesiologists and echocardiographers to access the patient through the radiation shield. It can be characterized as a necessary and sufficient size.

Regarding simulation of the air absorbed dose distribution, compared to earlier reported dosimetry performed using X-ray fluoroscopy equipment such as the C-Arm, no marked discrepancy was found in the dose or the dose gradient with distance from the tube [11–13]. Unlike surgeons and echocardiographers who perform TEE monitoring, anesthesiologists move constantly around the operating room. For that reason, although individual dosimetry in personal dosimetry measurements can indicate the actual exposure dose of anesthesiologists, it is difficult to use such information for protection. Estimating the anesthesiologists’ doses from dose measurements or exposure simulations at a single point in the operating room is also difficult. This study demonstrated that differences in exposure doses at each site, depending on the irradiation direction, are an important finding. An air dose distribution for the entire operating room is also assumed to be created as in this study. In that case, it is theoretically possible to estimate the exposure dose by combining the anesthesiologist escape behavior during irradiation in accordance with the fluoroscopic conditions and tracking of the anesthesiologist.

Monitoring the patient’s general condition during an operation is an important task for the anesthesiologist, in addition to the desirable practice of avoiding a high dose range during irradiation. However, patients undergoing SHD catheterization are often elderly people who are in poor general condition. Moreover, strict respiratory and circulatory management often requires medications and other procedures to be continued near the patient’s head. In such cases, if a radiation shield with both workability and shielding, such as our perforated shield, could be installed in addition to personal protective equipment, then the patient could be managed more safely with personnel near the patient than without the shield.

Although their work was conducted in the field of pediatric anesthesiology, Whitney *et al.* reported in 2019 a survey of pediatric anesthesiologists in the United States. Their findings indicated that 72.7% of physicians were concerned or very concerned about radiation exposure in their daily practice. The degree of concern was not reflective of the rate of personal dosimeter wearing or use of personal protective equipment. Furthermore, only 58.5% of anesthesiologists responded that they always or often use mobile or fixed lead acrylic plates [14]. These results suggest that anesthesiologists face an apparent paradox: they are concerned about radiation exposure but they are not thorough about protection. Results of this study also suggest a quandary, which demands promotion of a correct understanding of radiation exposure and adequate protection.

A review of radiation doses to the lens of the eye was presented in ICRP Publication 118. In Japan, earlier reports have described that it is appropriate to set the equivalent dose limit for the lens at an average of 20 mSv/year for five years; moreover, it must not to exceed 50 mSv/year in any one year. To achieve 20 mSv per year in the right lens of the perpendicular view, which has the highest absorbed dose rate, 1250 cases would be needed, assuming that each case is exposed to radiation at the patient’s head side for one hour. This is not realistically achievable. However, the fluoroscopic conditions in this study were set to the lowest dose rate at our institution. The radiation dose rate is almost proportional to the tube current and frame rate. Because the tube current and frame rate increase as a result of patient physique and other factors, the number of cases reaching the dose limit decreases as the radiation dose increases.

This study had several limitations. First, we selected the three irradiation angles which are generally recommended for the procedure currently performed at our hospital. We did not simulate other irradiation angles. These angles were chosen because irradiation from the specific angles of the PA projection, the Cusp-overlap projection and the Perpendicular projection accounts for most of the irradiation time in Procedures TEER, TAVR (self-expanding valves) and TAVR (balloon expanding valves). Therefore, it seems probable that the present simulation calculates the exposure to typical irradiation.

As might be apparent from the results obtained through this study, the skin dose to the anesthesiologist and the air dose distribution in the room differ greatly depending on the irradiation angle. However, in most situations, the proportion of time spent at angles other than the one selected in this study is negligible. Therefore, the effect on dose estimation is considered small.

Second, many other objects, such as C-arms and people, exist in an operating room. These objects are not included in this simulation because their complex shapes complicate the calculation. Moreover, their positions are not constant. Because it is unlikely that any large object other than a radiation shield will persist as a barrier between the tube sphere and the anesthesiologist, the dose to the anesthesiologist during the work is regarded as reasonable for the values used for the present simulation. If there is a shielding object between them, then the actual dose would be lower, so the present dose estimate is regarded as an estimate of the maximum value.

Third, anesthesiologists actually wear personal protective equipment during surgery. For that reason, the actual exposure at the body surface is expected to be lower. With a 0.25-mm lead apron, the transmission of a 60-kVp X-ray beam is reportedly 2–8%. That of a 90-kVp X-ray beam is 8–15% [15]. However, specifically regarding radiation safety goggles, anesthesiologists, who, unlike surgeons, move around a lot, are reluctant to wear them. However, if they are not worn, it would be better to install a protective plate like that used in the present study to prevent cataracts.

Fourth, we have only evaluated the radiation protection effects on the anesthesiologist’s side. We would like to evaluate the echocardiologist’s side when the device is put it into practical use.

Finally, we would like to evaluate the radiation shielding effects of our perforated radiation shield, not only through simulations but also using clinical data. This issue is expected to be addressed in future studies.

From computer simulations that we used to assess anesthesiologists’ exposure to radiation during SHD catheterization, the authors obtained knowledge of dose reduction effects of installing a perforated radiation shield that was devised to combine work efficiency and radiation protection.

### DATA AVAILABILITY

The data underlying this article are available in the article and in its online supplementary material.

## CONFLICT OF INTEREST

Dr. Kataoka has remuneration from Abbott Medical Japan as transesophageal echocardiography proctor for MitraClip.

## FUNDING

This work was supported by TERUMO LIFE SCIENCE FOUNDATION; Japan 2020 R&D subsidies; Abbott Medical Japan LLC. scholarship fund; Boston Scientific Co. scholarship fund; a Japan Society for the Promotion of Science (JSPS) KAKENHI Grant [21K07656 and 22H05108] and JST ERATO Grant [JPMJER2102], Japan.

## Supplementary Material

supplemental_figure1_rrac106

supplemental_figure2_rrac106

supplemental_figure3_rrac106

supplemental_figure4_rrac106

supplemental_figure5_rrac106

supplemental_figure6_rrac106

supplemental_figure7_rrac106

supplemental_figure8_rrac106

Supplemental_Figure_Legends_rrac106
