# Supplementary figures and images for: New perforated radiation shield for anesthesiologists: Monte Carlo simulation of effects
Source: J Radiat Res. 2023 Jan 25;64(2):379–86. doi: 10.1093/jrr/rrac106 (PMC10036102; doi:10.1093/jrr/rrac106)

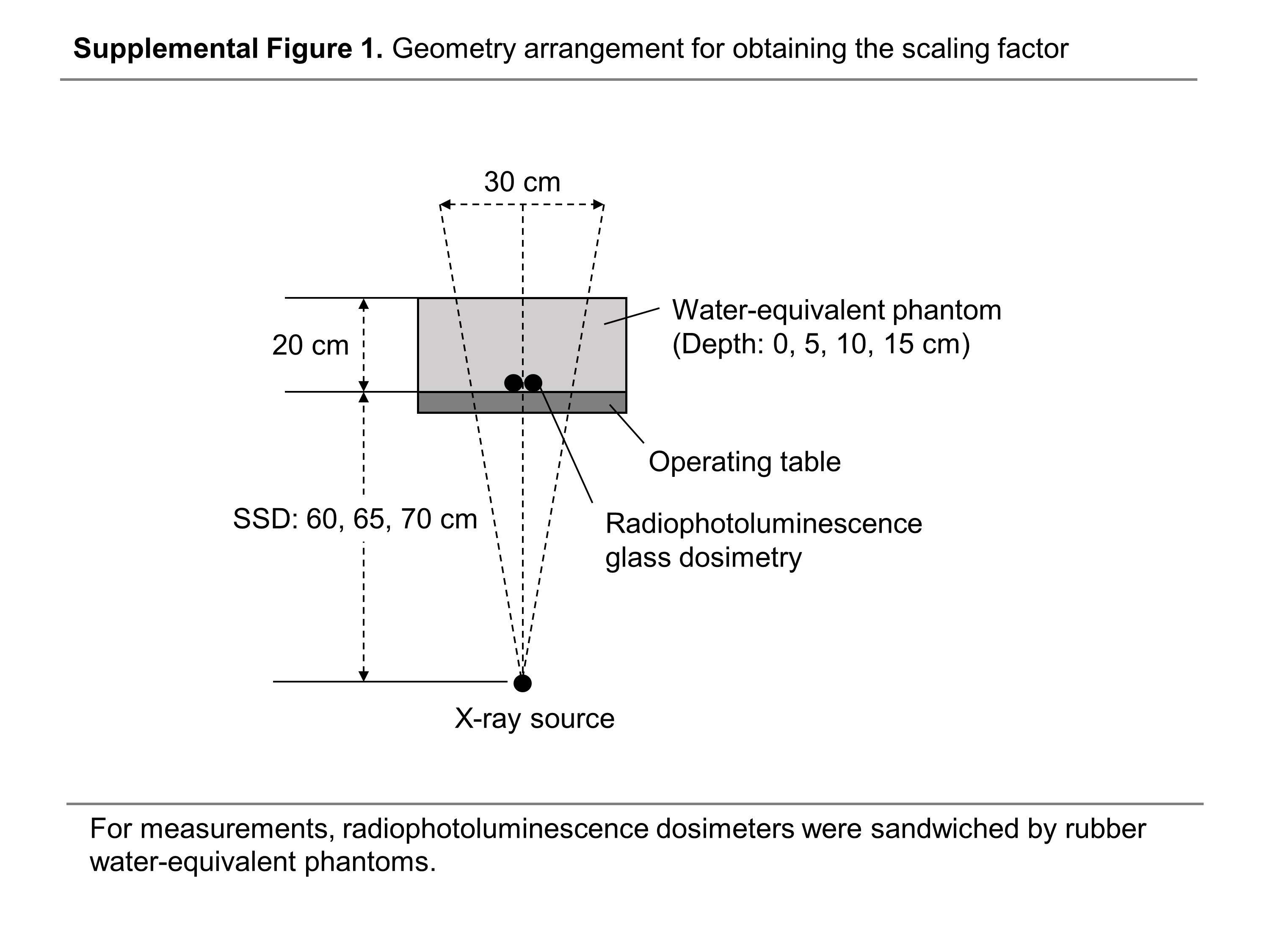

Supplement: supplemental_figure1_rrac106 [file supplemental_figure1_rrac106.jpeg]

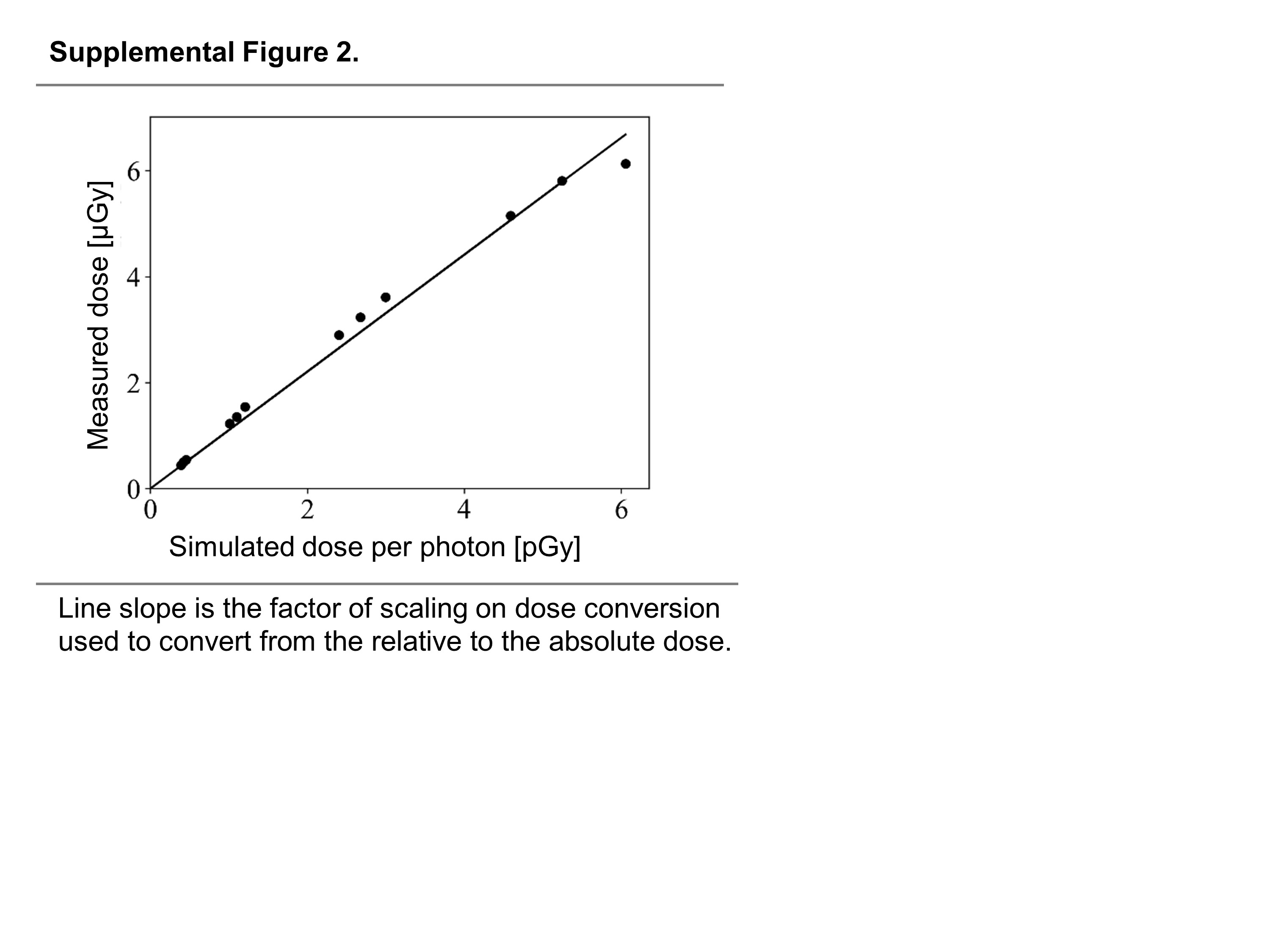

Supplement: supplemental_figure2_rrac106 [file supplemental_figure2_rrac106.jpeg]

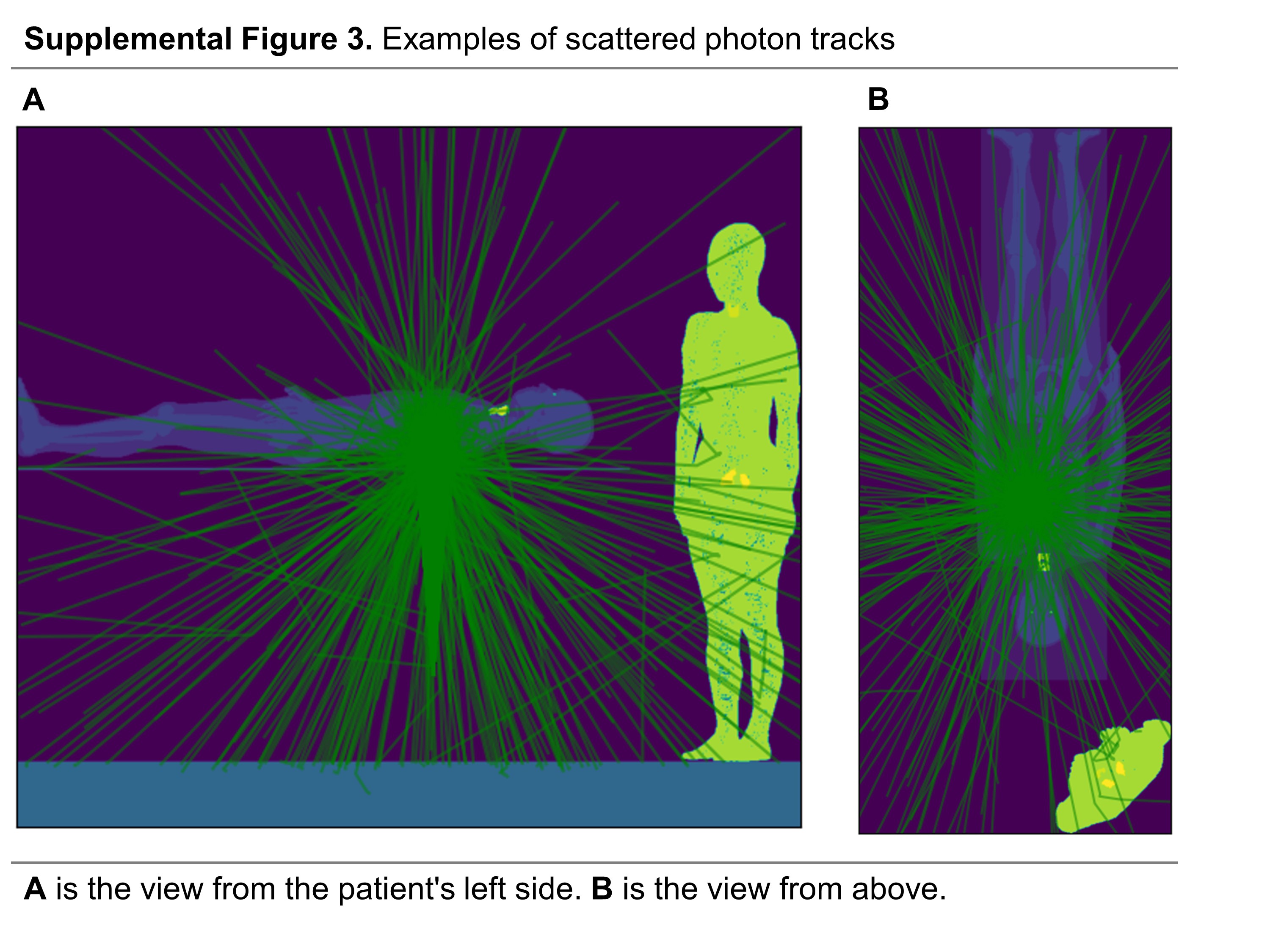

Supplement: supplemental_figure3_rrac106 [file supplemental_figure3_rrac106.jpeg]

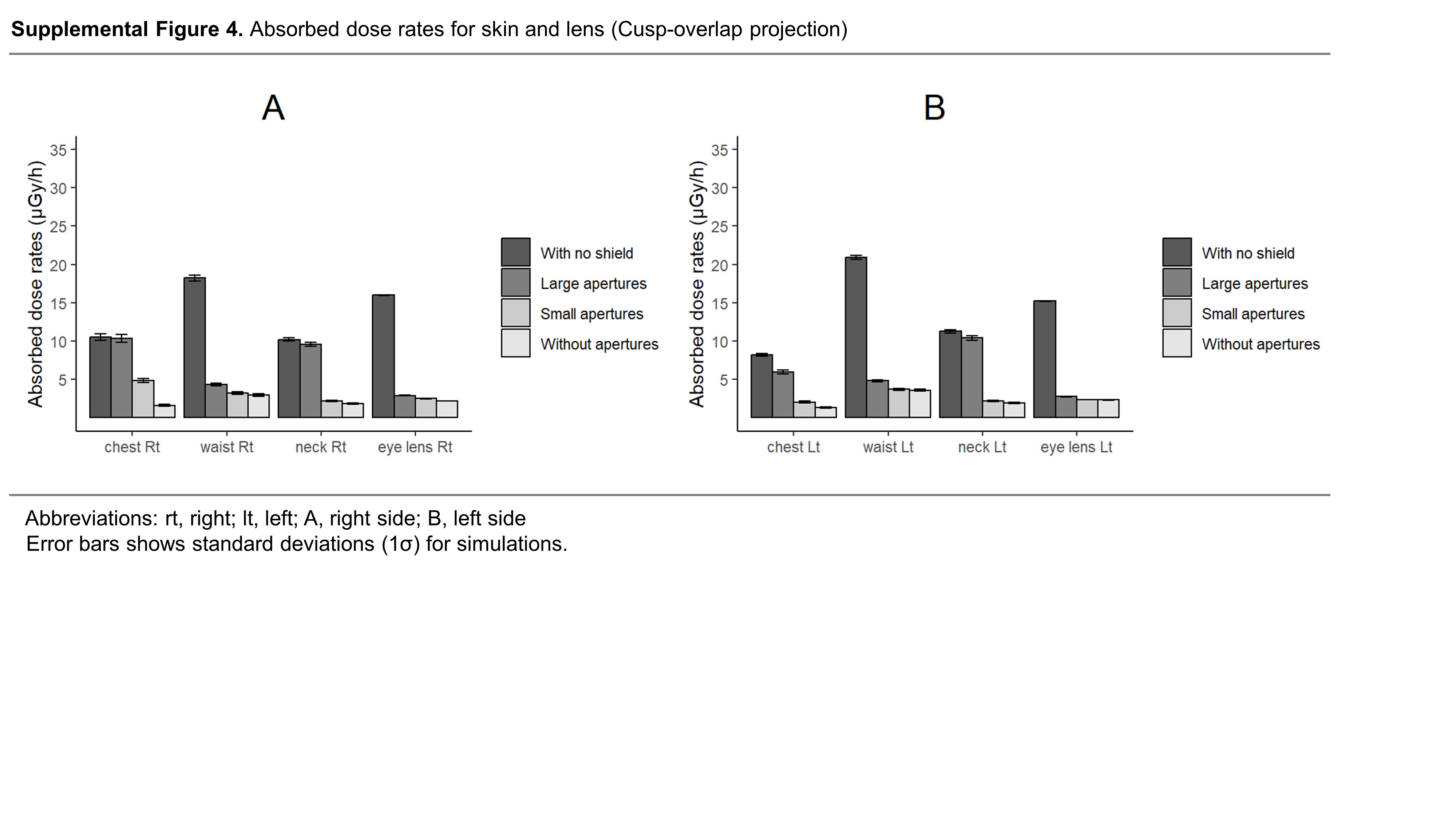

Supplement: supplemental_figure4_rrac106 [file supplemental_figure4_rrac106.jpeg]

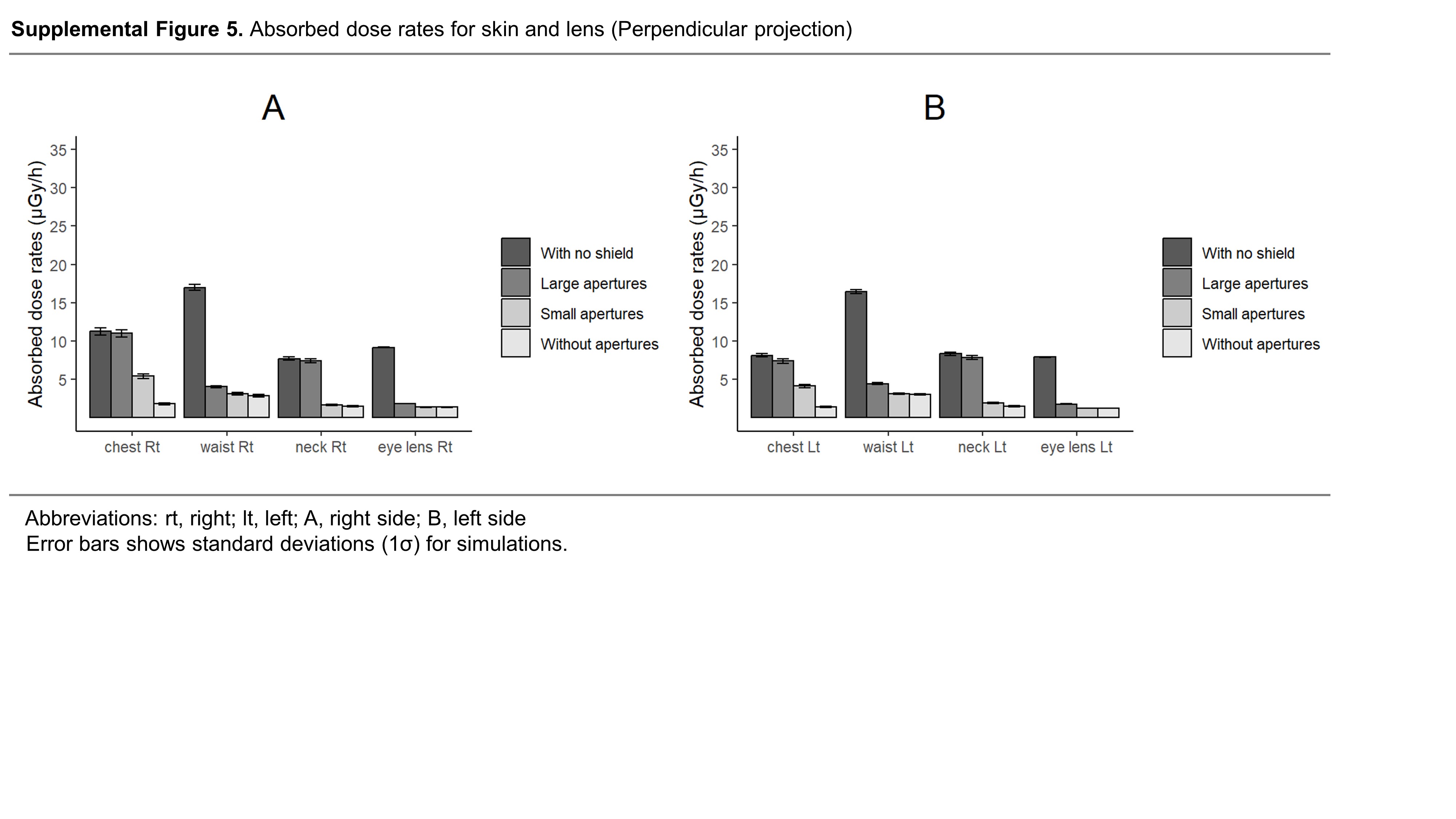

Supplement: supplemental_figure5_rrac106 [file supplemental_figure5_rrac106.jpeg]

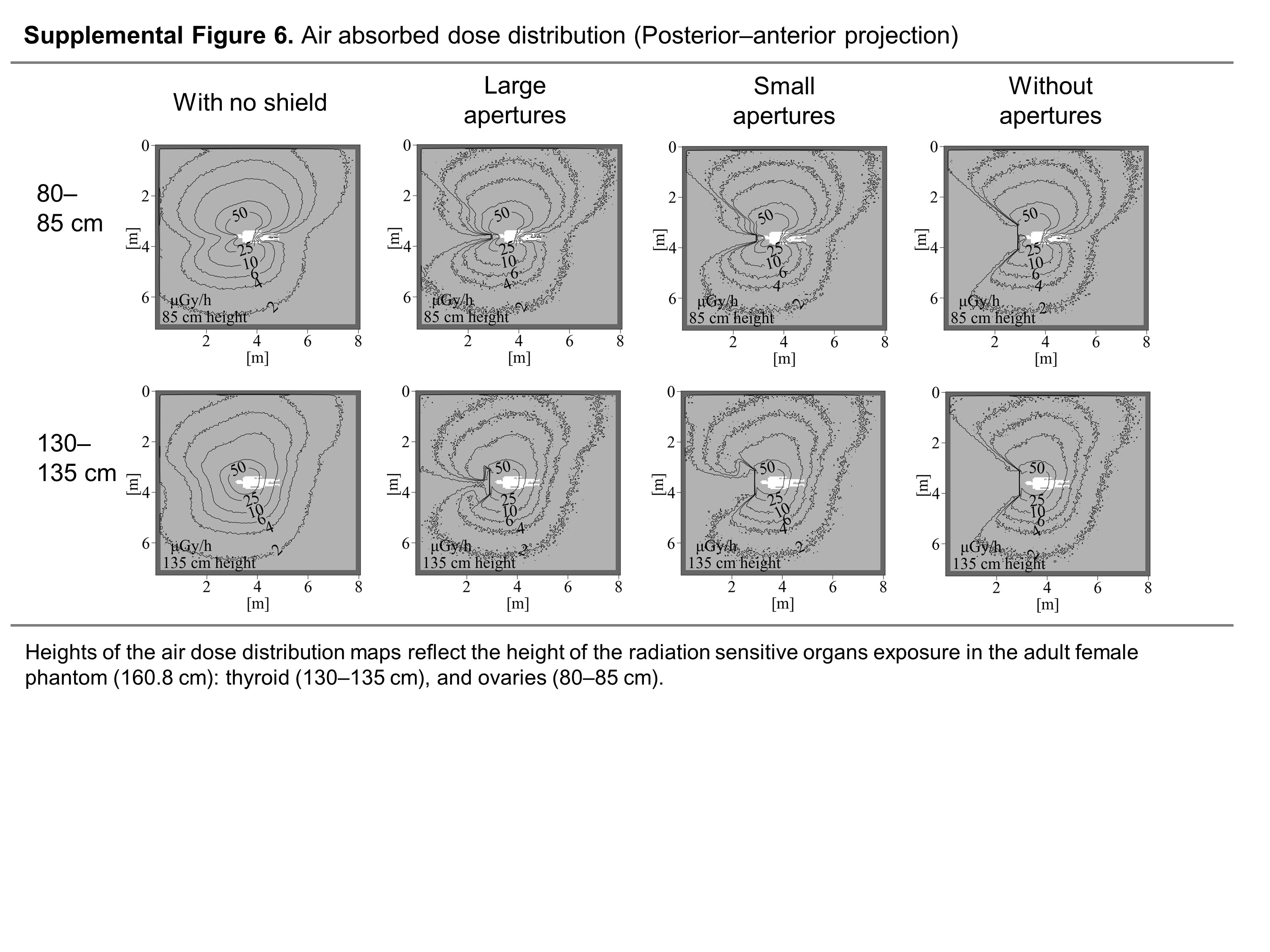

Supplement: supplemental_figure6_rrac106 [file supplemental_figure6_rrac106.jpeg]

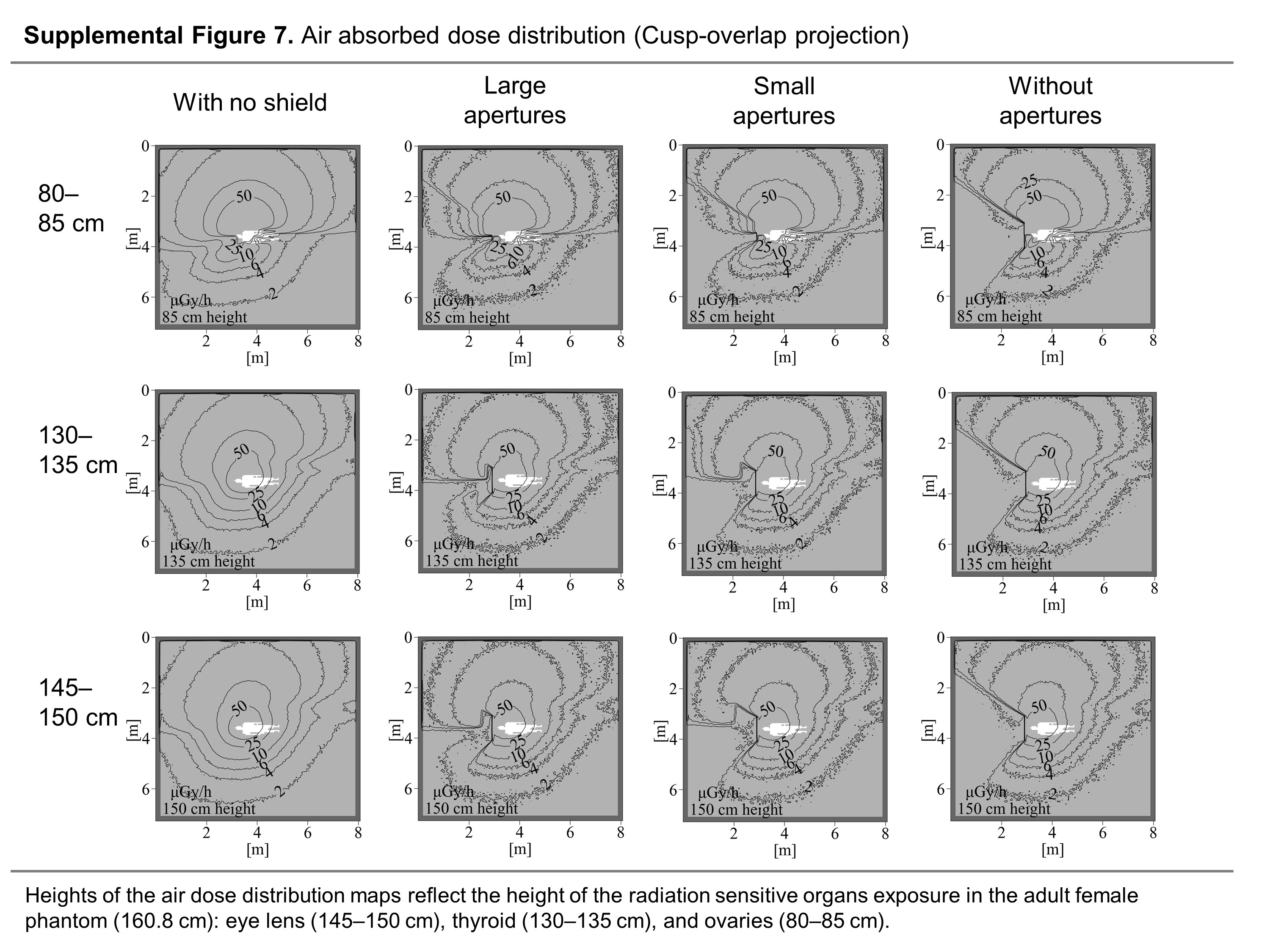

Supplement: supplemental_figure7_rrac106 [file supplemental_figure7_rrac106.jpeg]

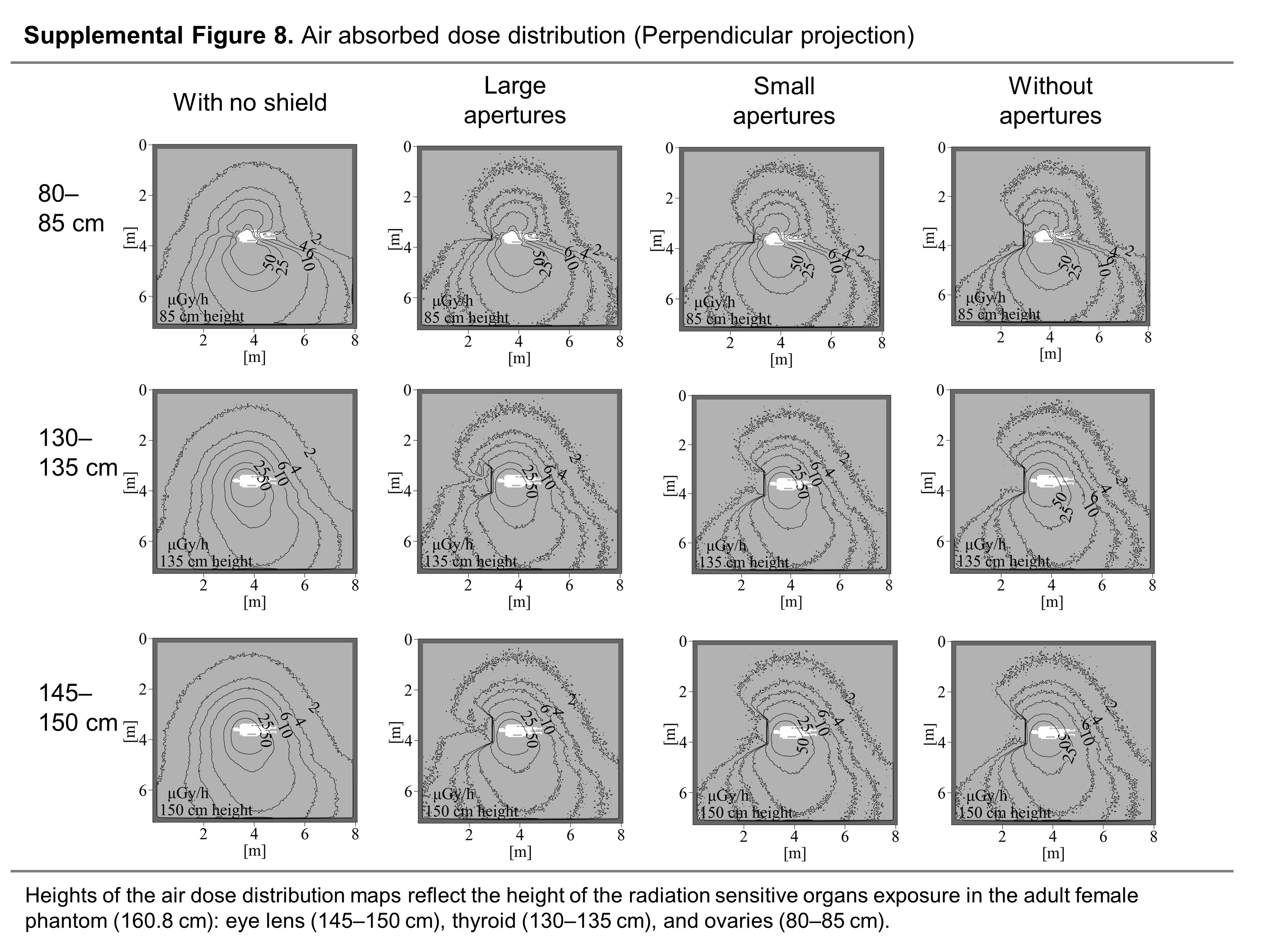

Supplement: supplemental_figure8_rrac106 [file supplemental_figure8_rrac106.jpeg]
