# Supplementary material for: New perforated radiation shield for anesthesiologists: Monte Carlo simulation of effects
Source: J Radiat Res. 2023 Jan 25;64(2):379–86. doi: 10.1093/jrr/rrac106 (PMC10036102; doi:10.1093/jrr/rrac106)
Supplement: Supplemental_Figure_Legends_rrac106 [file supplemental_figure_legends_rrac106.docx]

**Supplemental Materials**

Supplemental Figure 1. Geometry arrangement for obtaining the scaling factor. For measurements, radiophotoluminescence dosimeters were sandwiched by water-equivalent phantoms.

Supplemental Figure 2. Line slope is the factor of scaling on dose conversion to convert from the relative to absolute dose.

Supplemental Figure 3. Examples of scattered photon tracks; A is the view from the patient's left side. B is the view from above.

Supplemental Figure 4. Absorbed dose rates for skin and eye lens (Cusp-overlap projection): A is the right side. B is the left side. Error bars show standard deviations (1σ) for simulations.

Supplemental Figure 5. Absorbed dose rates for skin and eye lens (Perpendicular projection): A is the right side. B is the left side. Error bars show standard deviations (1σ) for simulations.

Supplemental Figure 6. Air absorbed dose distribution (Posterior–anterior projection). Heights of the air dose distribution maps reflect the height of the radiation-sensitive organ's exposure in the adult female phantom (160.8 cm): thyroid (130–135 cm) and ovaries (80–85 cm).

Supplemental Figure 7. Air absorbed dose distribution (Cusp-overlap projection). Heights of the air dose distribution maps reflect the height of the radiation-sensitive organ's exposure in the adult female phantom (160.8 cm): eye lens (145–150 cm), thyroid (130–135 cm), and ovaries (80–85 cm).

Supplemental Figure 8. Air absorbed dose distribution (Perpendicular projection). The heights of the air dose distribution maps reflect the height of the radiation-sensitive organ's exposure in the adult female phantom (160.8 cm): eye lens (145–150 cm), thyroid (130–135 cm), and ovaries (80–85 cm).
